# Hydrogen Sulfide Ameliorates Developmental Impairments of Rat Offspring with Prenatal Hyperhomocysteinemia

**DOI:** 10.1155/2018/2746873

**Published:** 2018-11-12

**Authors:** O. V. Yakovleva, A. R. Ziganshina, S. A. Dmitrieva, A. N. Arslanova, A. V. Yakovlev, F. V. Minibayeva, N. N. Khaertdinov, G. K. Ziyatdinova, R. A. Giniatullin, G. F. Sitdikova

**Affiliations:** ^1^Kazan Federal University, Kazan 420008, Russia; ^2^Kazan Institute of Biochemistry and Biophysics, FRC Kazan Scientific Center of RAS, Kazan 420011, Russia; ^3^A.I. Virtanen Institute, University of Eastern Finland, Kuopio 70211, Finland

## Abstract

Maternal high levels of the redox active amino acid homocysteine—called hyperhomocysteinemia (hHCY)—can affect the health state of the progeny. The effects of hydrogen sulfide (H_2_S) treatment on rats with maternal hHCY remain unknown. In the present study, we characterized the physical development, reflex ontogeny, locomotion and exploratory activity, muscle strength, motor coordination, and brain redox state of pups with maternal hHCY and tested potential beneficial action of the H_2_S donor—sodium hydrosulfide (NaHS)—on these parameters. Our results indicate a significant decrease in litter size and body weight of pups from dams fed with methionine-rich diet. In hHCY pups, a delay in the formation of sensory-motor reflexes was observed. Locomotor activity tested in the open field by head rearings, crossed squares, and rearings of hHCY pups at all studied ages (P8, P16, and P26) was diminished. Exploratory activity was decreased, and emotionality was higher in rats with hHCY. Prenatal hHCY resulted in reduced muscle strength and motor coordination assessed by the paw grip endurance test and rotarod test. Remarkably, administration of NaHS to pregnant rats with hHCY prevented the observed deleterious effects of high homocysteine on fetus development. In rats with prenatal hHCY, the endogenous generation of H_2_S brain tissues was lower compared to control and NaHS administration restored the H_2_S level to control values. Moreover, using redox signaling assays, we found an increased level of malondialdehyde (MDA), the end product of lipid peroxidation, and decreased activity of antioxidant enzymes such as superoxide dismutase (SOD) and glutathione peroxidase (GPx) in the brain tissues of rats of the hHCY group. Notably, NaHS treatment restored the level of MDA and the activity of SOD and GPx. Our data suggest that H_2_S has neuroprotective/antioxidant effects against homocysteine-induced neurotoxicity providing a potential strategy for the prevention of developmental impairments in newborns.

## 1. Introduction

Homocysteine, a sulfur-containing amino acid, is an intermediate product of the methionine metabolism. The concentration of homocysteine is regulated by remethylation back to methionine by methionine synthase, using 5-methyl tetrahydrofolate as cosubstrate that requires folic acid, or it can be catabolized by cystathionine *β*-synthase (CBS), a vitamin B6-dependent enzyme, to form cysteine and hydrogen sulfide (H_2_S) [1]. In humans, an increase of total plasma homocysteine to a level more than 15 *μ*M is defined as hyperhomocysteinemia (hHCY). According to the total plasma homocysteine level, it is classified as mild (15–25 *μ*M), moderate (25–50 *μ*M), or severe (50–500 *μ*M) hHCY [2]. hHCY may be induced by an increase of methionine in the diet, vitamin deficiency (folate, B12, or B6), mutations of genes encoding methylene tetrahydrofolate reductase (MTHFR), limiting the cells methylating capacity, or CBS [3]. hHCY is a risk factor of cardiovascular diseases, associated with cognitive impairments, increased risk of Alzheimer's disease, vascular dementia, or cerebrovascular stroke [4]. An elevated level of homocysteine is associated with common pregnancy complications such as pregnancy-induced hypertension, placenta abruptio, thromboembolic events, neural tube defects, and intrauterine growth restriction. Infants born from mothers with hHCY exhibit mental and physical retardation [1, 5]. In animal models, maternal hHCY induced oxidative stress and apoptosis in the fetal brain, resulting in postnatal neurodevelopmental deficits [6–10].

H_2_S is a one of the metabolites of homocysteine produced by CBS and cystathionine *γ*-lyase (CSE), enzymes of the transulfuration pathway of methionine metabolism [11]. In addition to the role of H_2_S as an important neuromodulator [12–14], H_2_S elicits neuroprotection against oxidative stress, neuroinflammation, apoptosis, and neurodegeneration caused by several pathophysiological conditions [15–17]. H_2_S donors attenuated lipopolysaccharide- or stress-induced learning and memory impairments in rats and prevented hippocampal long-term depression (LTD) [18, 19].

Altered H_2_S signaling was suggested to contribute in homocysteine-induced neurotoxicity [20, 21]. Indeed, intracerebroventricular administration of homocysteine decreased CBS expression and endogenous H_2_S generation in the hippocampus of rats along with learning and memory dysfunctions [22, 23, 24]. The results indicate that H_2_S is effective in providing protection against neurodegeneration and cognitive dysfunctions in homocysteine exposed rats. Nevertheless, the effects of H_2_S treatment on rats with maternal hHCY remain unknown. Current therapies for hHCY are limited to vitamin supplements, which serve as cofactors in the pathways of homocysteine metabolism. These therapies lower the level of homocysteine but generally do not alter disease consequences [11]. In the present study, we (1) evaluated the developmental consequences of maternal hHCY in rats; (2) assessed the effects of treatment with the H_2_S donor during pregnancy on physical parameters, neurobehavioral reflexes, muscle strength, and motor balance of the offspring; (3) evaluated the level of H_2_S and the rate of H_2_S generation in brain tissues of rats from control, hHCY, and NaHS-treated groups; (4) compared the oxidative stress level in brain tissues of pups born from the dams of control, hHCY, and NaHS-treated groups by measuring the concentrations of malondialdehyde (MDA), the end product of lipid peroxidation, and the activity of the antioxidant enzymes—superoxide dismutase (SOD) and glutathione peroxidase (GPx).

## 2. Materials and Methods

### 2.1. Experimental Animals and the Model of hHCY

Experiments were carried out on Wistar rats in accordance with EU Directive 2010/63/EU for animal experiments and the Local Ethical committee KFU (protocol no. 8 from 5.05.2015). Animals were housed in polypropylene cages (32 × 40 × 18 cm) under controlled temperature (22–24°C), with a 12 : 12 L/D light schedule (lights on at 6:00 a.m.) and free access to food and water. Pregnant rats were divided into four groups as follows. One group was fed ad libitum with a control diet (*n* = 7); the second group (*n* = 11) received daily methionine (7.7 g/kg body weight) with food starting 3 weeks prior to and during pregnancy [10, 25]. The third group (*n* = 4) received NaHS three weeks before and throughout pregnancy according the following protocol: 7 days of injections alternated with 7 days of adaptation. Rats of the fourth group (*n* = 4) received daily methionine and injections of NaHS according the abovementioned protocols. NaHS was used as the H_2_S donor and was diluted in sterilized saline and injected subcutaneously (i.s.c.) at a dose 3 mg/kg.

The offspring was divided into the following groups according to maternal diet: (1) control diet group (*n* = 61 pups/7 dams/7 litters), (2) methionine diet group (Hcy, *n* = 85 pups/11 dams/11 litters), (3) control diet group receiving NaHS (H_2_S, *n* = 54 pups/4 dams/4 litters), and (4) methionine diet group receiving NaHS (HcyH_2_S, *n* = 54 pups/4 dams/4 litters).

### 2.2. Maturation of Physical Features

After delivery, the litter size, total litter weight, and weight of each pup were assessed. Body weight was measured daily using an electronic balance (Vibra, model AJ-1200CE, Japan). Mortality was calculated as percent of dead pups against all pups in a litter during the observation period (P2–P28). The analysis of the physical development and reflex ontogeny was started at P2 and was carried out daily between 12 and 17 p.m. until P28 according to the previous studies [10, 26, 27]. The following physical features were observed: eye opening, ear unfolding, incisor eruption, and hair appearance. The maturation age of a particular feature was defined as the day on which that features were observed for the first time.

### 2.3. Reflex Testing

The time of appearance of each reflex was defined as the first day of its occurrence (Table 1) [27]. The following reflexes were scored: negative geotaxis, head shake, righting, cliff avoidance, acoustic startle reflex, cliff avoidance caused by visual stimulus, free-fall righting, and olfactory discrimination [10, 27].

### 2.4. Open Field Test

Rats were subjected to an open field test at P8, P16, and P26. The apparatus used to measure locomotion and exploratory activity was a round arena 0.3 m in diameter for P8 pups and 0.6 m for P16 and P26 pups with a floor divided into 36 parts and walls 0.1 and 0.2 m high, correspondingly (Open Science, Moscow, Russia). P8 animals were placed in the middle of the open field for 1 min and P16 and P26 animals for 3 min. The following parameters were evaluated: the number of crossings, head rearings, rearings, exploratory activity, grooming episodes, and defecation scores. After each experimental session, the arena was cleaned with a 0.5% ethanol solution.

### 2.5. Muscle Endurance

Muscle endurance was assessed by the paw grip endurance (PaGE) test [28] at P4, P14, and P26. Rats were placed on a wire grid and gently shaken to prompt the rat to grip the grid. The lid was turned upside down over a housing cage and held at ~0.45 m above an open cage bottom. The time (s) spent on the grid before falling was assessed. The largest value from three individual trials was used for analysis.

### 2.6. The Rotarod Test

The rotarod test was used to assess the motor coordination of fore and hind limbs and balance at P16, P21, and P26 [29] using rotarod (Neurobotix, Russia). Each rat was placed on the rotating rod with a rotation speed of 5 rotations per min (rpm), and the time to fall off and the running distance were measured. Animals are subjected to three consecutive test sessions (trials) with an interval of 20–30 min. The best of the latency to fall off the rotating rod was recorded [30].

### 2.7. Assay for Homocysteine Concentration and H_2_S synthesis

The total homocysteine level in plasma was determined by voltammetric measurements of products of the reaction with о-quinone [10, 31].

H_2_S synthesis assay was carried out using the N,N-dimethyl-p-phenylenediamine sulphate (NNDPD) method [32]. Brain tissues of rats (P28) were homogenized in ice-cold 0.15 M NaCl with phosphate buffer. The homogenate (10%, 860 *μ*l) was mixed with zinc acetate (1%, 500 *μ*l) and saline (140 *μ*l) at room temperature. Trichloroacetic acid (10%, 500 *μ*l) was added to precipitate proteins and stop the reaction. NNDPD (20 mM, 266 *μ*l) in 7.2 M HCl and FeCl_3_ (30 mM, 266 *μ*l) in 1.2 M HCl were added to the mixture, and absorbance of resulting solution (600 *μ*l) was measured by a spectrophotometer at 670 nm (PE-5300VI, ECOHIM, Russia).

H_2_S generation rate was measured in a mixture containing homogenate (10%, 860 *μ*l), L-cysteine (10 mM, 40 *μ*l), pyridoxal 5′-phosphate (2 mM, 40 *μ*l), and saline (60 *μ*l). After incubation at 37°C for 60 min, zinc acetate (1%, 500 *μ*l) was injected to trap the produced H_2_S followed by trichloroacetic acid (10%, 500 *μ*l) addition. Then, NNDPD (20 mM, 266 *μ*l) in 7.2 M HCl and FeCl_3_ (30 mM, 266 *μ*l) in 1.2 M HCl were added, and absorbance of aliquots of the resulting solution (600 *μ*l) was measured at 670 nm by a spectrophotometer. H_2_S concentration was calculated against a calibration curve of NaHS, and H_2_S synthesizing activity is expressed as *μ*M H_2_S produced by 1 g tissue per minute (*μ*M/min/g).

### 2.8. Lipid Peroxidation and the Activity of SOD and GPx

Malondialdehyde (MDA) was measured using a spectrophotometer according to the method of Ohkawa et al. [33]. Samples of brain tissue were fixed in liquid nitrogen, then homogenized and mixed at a ratio 1 : 1 with 0.3% Triton Х-100, 0.1 М НС1, and 0.03 М 2-thiobarbituric acid (ТBA). The mixture was heated for 45 min at 95°C and centrifuged for 10 min at 10,000*g*. Under this condition, MDA readily participates in a nucleophilic addition reaction with 2-thiobarbituric acid (TBA), generating a red, fluorescent 1 : 2 MDA adduct. The absorbance of the supernatant was monitored at 532 nm and at 560 nm (*ε*_TBA-MDA_ = 1.55 mM^−1^ cm^−1^). MDA levels were expressed as *μ*g/g of tissues.

The antioxidant potential was determined by measuring activities of glutathione peroxidase (GPx) and superoxide dismutase (SOD). Samples of brain tissue were fixed in liquid nitrogen, homogenized in cold buffer solution (0.1 M MES at pH 6.0, ratio 1 : 10), and centrifuged for 10 minutes at 10,000*g*. SOD activity (Cu/Zn superoxide dismutase) was determined according to Weyder and Cullen [34]. Applying this method, a xanthine/xanthine oxidase system was used to generate O_2_^·−^ and nitroblue tetrazolium (NBT) reduction was used as an indicator of O_2_^·−^ production. SOD competes with NBT for O_2_^·−^. The percent inhibition of NBT reduction reflects the amount of SOD which is assayed using a spectrophotometer at 560 nm. The reaction mixture contained 100 mM Na_2_HPO_4_ buffer (pH 10.2), 0.1 mM EDTA, 1 M cytochrome *c*, 1 mM xanthine, 0.04 mM NBT, and 150 *μ*l of the sample. The reaction was initiated by the addition of 0.05 unit of xanthine oxidase. The inhibition of the produced chromogen is proportional to the activity of the SOD present in the sample. A 50% inhibition is defined as 1 unit of SOD, and specific activity is expressed as units per milligram of protein (U_SOD_/min/mg).

GPx activity was also determined according to Weyder and Cullen [34]. GPx catalyzes the oxidation of glutathione by cumene hydroperoxide. In the presence of glutathione reductase and NADPH, the oxidized glutathione (GSSG) is quickly converted to the reduced form with a concomitant oxidation of NADPH to NADP^+^. The decrease in absorbance was monitored with a spectrophotometer at 340 nm. The reaction mixture consisted of 50 mM Na_2_HPO_4_ buffer (pH 7.2), 1 mM reduction glutathione (GSH), 0.5 unit of glutathione reductase, 0.15 mM NADPH, 1 mM EDTA, and 150 *μ*l of the sample. One GPx unit is defined as 1 *μ*mol of GSH consumed per minute, and the specific activity is reported as units per mg of protein (U_POX_/min/mg).

Protein content was measured using Bradford's assay [35] employing bovine serum albumin as standard. A volume of 20 *μ*l of the sample or standard was mixed with a 1 ml Bradford reagent, and the absorbance was assessed by a spectrophotometer at 595 nm after 5 min.

### 2.9. Statistical Analysis

Normality of the sample data was evaluated with the Shapiro-Wilk test (sample size less than 25) or Kolmogorov-Smirnov test (sample size more than 25) for equal variances using *F*-test Origin Pro software (OriginLab Corp., Northampton, MA, USA). Data are expressed as median (Q1–Q3) or mean ± SEM. Statistical significance between medians was calculated using the nonparametric ANOVA Kruskal-Wallis test and Mann-Whitney test in Origin Pro 2015 (OriginLab Corp., USA). Statistical significance between means was calculated using parametric one-way ANOVA followed by the Bonferroni test in Origin Pro 2015 (OriginLab Corp., USA). Differences were considered as statistically significant at *p* < 0.05 (for parametric test) and Pu < 0.05 (for nonparametric test); *n* indicates the number of animals.

## 3. Results

### 3.1. Maturation of Physical Features

**Figure 1:**
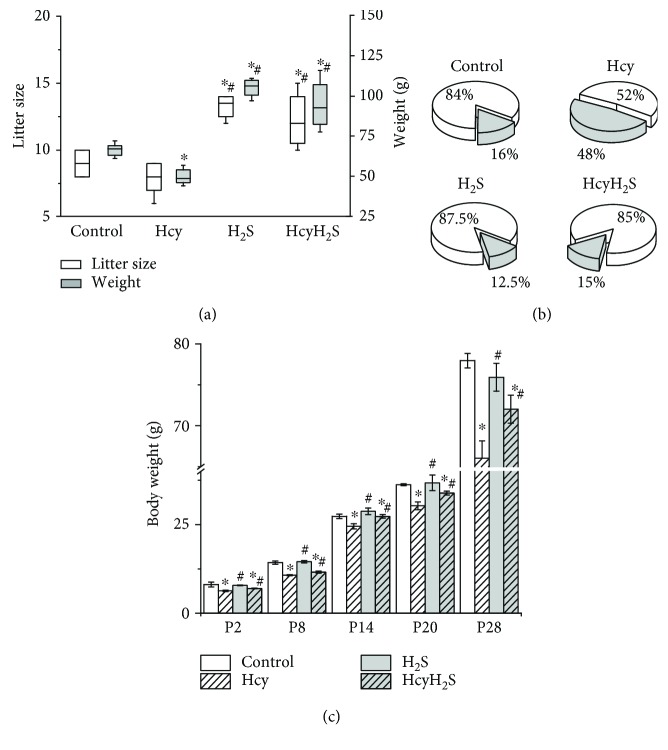
Effects of maternal hyperhomocysteinemia and NaHS treatment on the litter size, litter weight, mortality, and weight gain of the offspring. (a) Box plots reflecting the litter size (white boxes) and litter weight (grey boxes) in the control, Hcy, H_2_S, and HcyH_2_S groups. (b) Mortality of pups during period P2–P28 in the control, H_2_S, Hcy, and HcyH_2_S groups. White part—alive pups, grey part—dead pups in % relatively the litter size. (c) Body weights of rat dams during the period P2–P28 from the control, H_2_S, Hcy, and HcyH_2_S groups. ^∗^*p* < 0.05 compared to the control group; ^#^*p* < 0.05 compared to the Hcy group.

The average litter size of control and Hcy groups at P0 did not differ significantly (8.6 ± 1.2 vs. 8.4 ± 1.6 pups in the Hcy group, Pu > 0.05). However, in H_2_S and HcyH_2_S groups, the average litter size was significantly higher (13.3 ± 1.5 and 13.5 ± 1.0, correspondingly, Pu < 0.05) (Figure 1(a)). At the same time the total litter weight was significantly lower in the Hcy group due to the low body weight of the pups (Figure 1(a)). In H_2_S and HcyH_2_S groups, the total litter weight was higher compared to the control and Hcy groups due to larger litter sizes (Figure 1(a)). Substantial growth retardation of pups from the Hcy group was recorded during all observation periods (P0–P28) (Figure 1(c)).

At P28, body weight was reduced from 79.9 ± 0.8 g in controls to 66.1 ± 2.1 g (*n* = 53) in deficient animals (*n* = 53, *p* < 0.05). The average body weight of pups in the Hcy and HcyH_2_S groups at P2 was significantly lower than in the control and H_2_S groups. However, beginning from P8, the weight gain of Hcy pups was lower compared to all other groups during the observation period (Figure 1(c)). The mortality of pups in the Hcy group was higher (48%) compared to the control group (16%). The mortality of pups in the H_2_S and HcyH_2_S groups did not differ from the control (Figure 1(b)). Other parameters of physical maturation such as ear unfolding, the primary hair appearance, incisor eruption, and eye opening were not different in all experimental groups.

### 3.2. Reflex Testing

We studied reflex ontogeny (righting reflex, negative geotaxis, cliff avoidance, head shake, acoustic startle reflex, free-fall righting, cliff avoidance caused by visual stimulus, and olfactory discrimination) reflecting brain maturation and integrity of sensorimotor development [26] (Table 1). Almost all reflexes were impaired in the Hcy group. Namely, negative geotaxis formation was delayed in the Hcy group (Table 1). The head shake reflex started at P8 in rat pups of all groups, but the number of head rotations per min was significantly lower in the Hcy group compared to the control, H_2_S, and HcyH_2_S groups (Table 1). In the Hcy group, the onset of the righting reflex was delayed and the time necessary to come back to a quadruped position was significantly increased compared to other groups (Table 1). In the pups of the Hcy group, the cliff avoidance reflex was formed later (at P7) compared to the control, H_2_S, and HcyH_2_S groups (Table 1). The delay of the reflex onset was also observed in pups of the Hcy group in other sensorimotor tests (Table 1).

### 3.3. Locomotion and Exploratory Activity in the Open Field Test

**Figure 2:**
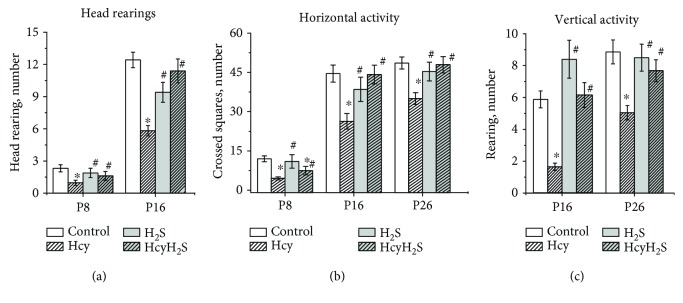
Effects of maternal hyperhomocysteinemia and NaHS treatment on locomotion in the open field test. Head rearings (a), the number of crossed squares (b), rearings (c) of pups from the control Hcy, H_2_S, and HcyH_2_S groups. Data are expressed as mean ± SEM. ^∗^*p* < 0.05 compared to the control group; ^#^*p* < 0.05 compared to the Hcy group.

The locomotor and exploratory activity was studied in the open field test at the ages P8, P16, and P26. Head rearing was analyzed in pups of P8 and P16. At P8 and P16, the number of head rearings in pups of the Hcy group was decreased compared to that in control, and in the H_2_S and HcyH_2_S groups, this parameter did not differ from the control (Figure 2(a)).

**Figure 3:**
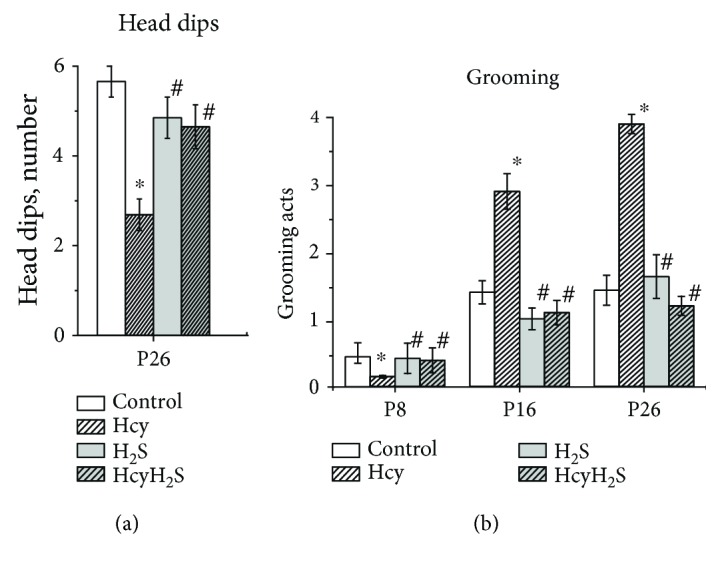
Effects of maternal hyperhomocysteinemia and NaHS treatment on the exploratory activity and emotionality in the open field test. Head dips (a) and grooming acts (b) of pups from the control Hcy, H_2_S, and HcyH_2_S groups. Data are expressed as mean ± SEM. ^∗^*p* < 0.05 compared to the control group; ^#^*p* < 0.05 compared to the Hcy group.

Horizontal activity was significantly lower in the Hcy group compared to the control group at all studied ages (Figure 2(b)). Administration of NaHS increased this parameter in pups with prenatal hHCY compared to the Hcy group. The number of crossed squares of pups from the H_2_S group was not different from the control group at all ages (Figure 2(b)). Rearings or vertical activity of pups from the Hcy group was significantly lower compared to the control. Activity of pups from the H_2_S and HcyH_2_S groups was higher compared to pups from the Hcy group (Figure 2(c)). Exploratory activity was assessed by the number of head dips at P26 (Figure 3(a)). The number of head dips from the Hcy group was significantly lower than in the control, the H_2_S, and HcyH_2_S groups (Figure 3(a)). Grooming behavior and defecation scores were used as a measure of emotionality of animals [30, 36]. No significant intergroup difference was found in scores of defecation, but in animals of the Hcy group, higher numbers of grooming episodes were observed at P16 and P26 and were significantly decreased at P8, probably reflecting the deficit of motor coordination and locomotor activity (Figure 3(b)).

### 3.4. Rotarod Test and the Paw Grip Endurance (PaGE)

**Figure 4:**
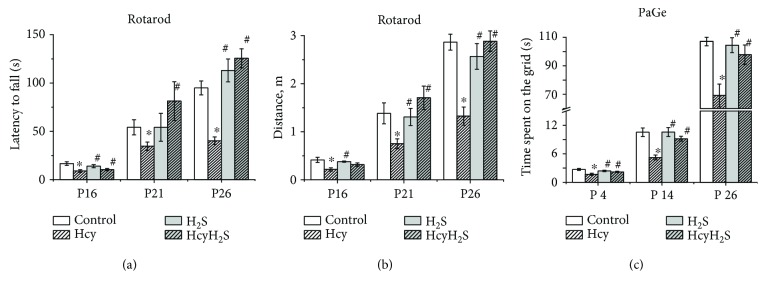
Effects of maternal hyperhomocysteinemia and NaHS treatment on muscle strength and motor coordination. Latency to fall (a) and running distance (b) in the rotarod test; the time spent on the grid (before falling) (c) in the paw grip endurance (PaGE) test of pups from the control Hcy, H_2_S, and HcyH_2_S groups. Data are expressed as mean ± SEM. ^∗^*p* < 0.05 compared to the control group; ^#^*p* < 0.05 compared to the Hcy group.

Motor coordination was assessed using the rotarod test, where the time to fall off and running distance were measured [29]. A significant reduction of the time spent on the rotarod was observed in the Hcy group at all age groups compared to the control (Figure 4(a)). Similar changes were also observed for the rotarod distance during experimental sessions for all studied ages (Figure 4(b)). NaHS treatment restored both parameters of the Hcy groups to control values.

In the control group, the time rats were able to stay on the grid increased with aging from 2.63 ± 0.36 s at P4 to 107.12 ± 7.46 s at P26 (Figure 4(c)). Rats from the Hcy group exhibited a deficit in the PaGE task as indicated by the reduction of time spent on the grid relatively to control rats (Figure 4(c)). NaHS treatment increased the time spent on the grid in pups of the Hcy group (Figure 4(c)).

### 3.5. Plasma Hcy Level

The concentration of homocysteine in the plasma in control females was 8.16 ± 0.29 *μ*M (*n* = 7) and in females fed with methionine-containing diet was 31.75 ± 2.18 *μ*M (*n* = 11). The concentration of homocysteine in the plasma of pups born from control animals was 6.23 ± 0.42 *μ*M (*n* = 32) and from females fed with methionine-containing diet was 22.07 ± 2.60 *μ*M (*n* = 32). These results indicate the development of hHCY in dams and their offspring. NaHS treatment did not induce any changes of homocysteine levels in dams (9.3 ± 0.6 *μ*M, *n* = 4) and pups (6.5 ± 0.3 *μ*M, *n* = 16) of the control group, however, significantly reduced concentration of homocysteine in dams with hHCY (17.4 ± 1.4 *μ*M, *n* = 4) and their offspring (17.1 ± 2.5 *μ*M, *n* = 16).

### 3.6. H_2_S Generation in Brain Tissues

It was shown previously that an exposure to homocysteine decreased the endogenous generation H_2_S in different tissues [21, 22, 24, 37]. In our experiments, H_2_S concentration, measured in brain tissues of control animals, was 12.76 ± 0.72 *μ*M (*n* = 7). In rats of the Hcy group, we observed the decrease of H_2_S concentration to 7.97 ± 0.87 *μ*M (*n* = 7, *p* < 0.05), which was elevated to 11.35 ± 2.01 *μ*M by NaHS administration in the HcyH_2_S group (*n* = 7). The activity of H_2_S-producing enzymes in the brain was measured as the rate of endogenous H_2_S generation when a high concentration of cysteine and pyridoxal 5′-phospate was added to brain homogenates. It was shown that the rate of H_2_S production decreased from 8.86 ± 1.24 *μ*M/min/g in the control (*n* = 7) to 2.84 ± 1.09 *μ*M/min/g in the Hcy group (*n* = 7, *p* < 0.05) and 2.25 ± 0.98 *μ*M/min/g in the HcyH_2_S group (*n* = 7, *p* < 0.05). Our data indicate that in rats with prenatal hHCY, the rate of endogenous generation of H_2_S brain tissues was lower than in control conditions and administration of NaHS to dams with hHCY increased the H_2_S level to the control values but did not restore the activity of H_2_S-producing enzymes.

### 3.7. Lipid Peroxidation and Antioxidant Enzymes Activity in Brain Tissues

**Figure 5:**
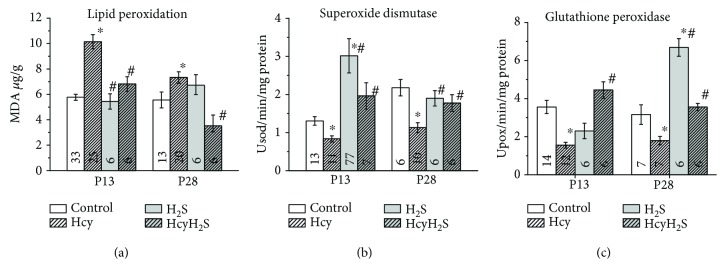
Effects of prenatal hHCY and NaHS treatment on lipid peroxidation and antioxidant enzyme activities measured in rat brain tissues. The level of MDA (an end product of lipid peroxidation) (a) and activities of antioxidant enzymes—superoxide dismutase 1 (b) and glutathione peroxidase 1 (c) measured in brain tissues of P13 and 28 rats from the control, Hcy, H_2_S, and HcyH_2_S groups. For each measurement, the number of samples is indicated inside the column. ^∗^*p* < 0.05 compared to the control group; ^#^*p* < 0.05 compared to the Hcy group.

Severe oxidative stress during the prenatal period induces neuroinflammation and apoptosis followed by retardation of fetal growth and developmental impairments in postnatal life [8]. In order to estimate the extent of the oxidative stress in rats with prenatal hHCY, the level of MDA was measured in brain tissues of P13 and P28 animals from the control, Hcy, H_2_S, and HcyH_2_S groups. At P13, the MDA level increased almost twice in the Hcy group which indicates a higher production of the reactive oxygen species (ROS) in rat brains with prenatal hHCY (Figure 5(a)). In rats of the HcyH_2_S group, the MDA level was significantly lower and did not differ from the control group (Figure 5(a)). In rats of the H_2_S group, the MDA level was not different from the control level (Figure 5(a)). Similar values were observed in P28 rats (Figure 5(a)).

It is well known that homocysteine induces oxidative stress by the production of intracellular superoxide radicals but also impairs the activity of antioxidant enzymes [38, 39]. Therefore, we analyzed the enzymatic activities of SOD and GPx in brain tissues from the control, Hcy, H_2_S, and HcyH_2_S groups. We found that the activity of SOD that converts superoxide anions into H_2_O_2_ was significantly lower in the group of P13 and P28 Hcy rats (Figure 5(b)). Namely, at P13, the SOD activity decreased the Hcy groups. In rats from the HcyH_2_S group, the SOD activity significantly increased and was not different from the control. Interestingly, in the H_2_S group, SOD activity was higher than both in the control and Hcy groups (*n* = 7, *p* < 0.05). At P28, the level of SOD activity in the Hcy group was almost half of the control group and NaHS treatment restored its activity (Figure 5(b)). Similarly, decreased activity of GPx which reduces peroxides was observed in the Hcy group of P13 and P28 animals and NaHS treatment restored its activity to control values (Figure 5(c)). Evidently, the imbalance of prooxidant and antioxidant systems during chronic exposure of the fetus to high concentrations of homocysteine caused an oxidative stress and functional disability in the postnatal period. At the same time, low doses of NaHS during pregnancy provided antioxidant protection during prenatal and early postnatal development.

## 4. Discussion

During pregnancy, several complications have been associated with elevated homocysteine levels including preeclampsia, placental abruption, intrauterine growth retardation, or neural tube defects [40]. Several studies demonstrated that maternal hHCY resulted in a deficit of learning and memory in the offspring due to delayed brain maturation [6–9]. In most of the previous studies, the analysis of behavior was performed with offspring at almost adult level [7], whereas the present study focused on the detailed analysis of the physical development and reflex ontogeny, exploratory activity, and motor coordination of pups during the first 3 weeks of development. Our results indicate a significant decrease in litter size and body weight and delay in the formation of sensorimotor reflexes of pups with maternal hHCY. Locomotor and exploratory activity tested in the open field was diminished in the pups of the Hcy group. Prenatal hHCY also resulted in reduced muscle strength and motor coordination deficits assessed by the paw grip endurance test and the rotarod test. Simultaneously, we observed an increased level of oxidative stress and decreased activity of the antioxidant enzymes—SOD and GPx—in brain tissues of pups with hHCY. In rats with prenatal hHCY, the endogenous generation of H_2_S brain tissues was lower than in control conditions. Administration of the H_2_S donor—NaHS—to dams with hHCY during pregnancy prevented the deleterious effects of high homocysteine levels on fetus development, lowered oxidative stress, increased the H_2_S level in brain tissues, and restored the activity of SOD and GPx indicating its antioxidant potential.

### 4.1. H_2_S Prevents Oxidative Stress and Decreases H_2_S Level in Brain Tissues of Rats with Prenatal hHCY

In the model of prenatal hHCY used in our study, female rats received high methionine diet before and during pregnancy which induced an elevation of the plasma homocysteine level four times compared to control values. High blood plasma levels of homocysteine were not only observed in dams with hHCY but also in their offspring according to previous data [41]. Indeed, homocysteine can be transferred successfully through the placental exchange barrier and fetal cord homocysteine concentrations related to the maternal level [41, 42, 43]. In fetal brain, homocysteine can be produced from methionine or can be transported through the blood-brain barrier [44]. Under these circumstances, the fetal development occurs in hHCY conditions, which results in high mortality, low litter size, and low body weight of the offspring as was shown in our present and several previous studies [6–10].

Placental pathology due to endothelial dysfunctions, impaired NO synthesis, oxidative stress, and inflammation underlies adverse pregnancy outcome during hHCY conditions [45]. Oxidative stress is one of the main mechanisms of homocysteine-induced neurotoxicity as during prenatal period ROS highly affect embryo and fetus due to the lack of adequate antioxidant protection [46, 47]. Homocysteine itself can undergo autooxidation of its free thiol groups binding via a disulfide bridges with plasma proteins, low molecular thiols, or with a second homocysteine molecule [39]. Indirect oxidative effects of hHCY include the generation of superoxide from xanthine oxidase or uncoupled endothelial nitric oxide synthase, downregulation of antioxidant enzymes, or depletion of intracellular glutathione [39, 48, 49]. ROS, produced in these reactions, further oxidize various functionally important proteins, lipids, and nucleic acids [50]. Indeed, in our experiments, we observed an increased level of MDA, reflecting a higher level of oxidative stress in rats with prenatal hHCY similar to previous data [6–9]. Moreover, we found decreased activity of the antioxidant enzymes—SOD and GPx—in brain tissues of rats with prenatal hHCY which results in augmented accumulation of ROS during hHCY conditions. The altered activity/expression of SOD and GPx was also shown in vitro and in vivo studies [51, 52] including brain samples of rats with hHCY [6, 7, 9, 36, 39].

Recent data indicate the contribution of endogenous H_2_S for healthy placental vasculature which provides placental perfusion and optimal oxygen and nutrient diffusion [53, 54]. Moreover, inhibition of CSE reduced placental growth factor production, induced hypertension, promoted abnormal labyrinth vascularization in the placenta, and decreased fetal growth [53]. At the same time, H_2_S donor treatment prevented these changes and improved pregnancy outcome [54]. In addition, an insufficient H_2_S level has been suggested to be one of the potential causes of oxidative stress [55] which in turn results in the reduction of placental CSE activity, decreased H_2_S production, and intrauterine fetal growth restriction [54]. Worth noting, low level of H_2_S and diminished rate of endogenous H_2_S generation in brain tissues of rats with prenatal hHCY were shown in our experiments. Interestingly, that administration of the H_2_S donor before and during pregnancy increased the concentration of H_2_S without affecting the activity of H_2_S-producing enzymes. H_2_S treatment not only restored the litter size and total litter weight of the offspring with maternal hHCY but even increased these parameters in control animals which appeared related to the improvement of placental blood supply and prevention of oxidative stress. Indeed, using spectrophotometric assays, we found that treatment with NaHS significantly lowered lipid peroxidation levels and restored the activity of SOD and GPx in brain tissues of rats with prenatal hHCY and even increased the activity of SOD and GPx in control animals. Positive effects of H_2_S were also shown in hHCY mice and rats where NaHS treatment attenuated oxidative stress, neurodegeneration, and neuroinflammation and restored the altered expression of synaptic proteins in hippocampal neurons and H_2_S level in brain tissues [16, 17, 23, 24]. Indeed, H_2_S with its reducing ability shows a high capacity to scavenge ROS [55]. H_2_S can react directly with superoxide anion (O_2_^−^), peroxynitrite, and other ROS [56]. Moreover, it was suggested that H_2_S can trigger antioxidant signaling pathways apart from its direct chemical reductant effect. Namely, H_2_S increases the level of two nonenzymatic antioxidants in animal cells, including intracellular reduced glutathione (GSH) and thioredoxin (Trx-1) [55, 57–59]. Mechanisms of H_2_S effects include the activation of the nuclear factor (erythroid-derived 2-) like 2 (Nrf2) and a transcription factor that regulates a wide variety of gene expression. Under oxidative stress conditions, Nrf2 is translocated into the nucleus and binds to promoters containing the antioxidant response element (ARE) sequence and inducing ARE-dependent gene expression such as Trx-1 and glutathione reductase [60–62].

H_2_S also increases the activity of enzymatic antioxidants like SOD, catalase, and GPx which is likely mediated by an upregulation of NF-*κ*B transcription factor [55, 63-65] or Nrf2 signaling cascade [66]. Moreover, H_2_S can directly bind at the catalytic Cu^2+^ center of SOD as a substrate, increases the rate of superoxide anion scavenging [63], and directly stimulates the activity of GPx in vitro and in vivo studies [55, 67].

### 4.2. H_2_S Accelerates the Development of Neurobehavioral Maturation, Improves Exploratory Behavior, and Decreases Anxiety of Rats with Prenatal hHCY

In rats, the period of two weeks after birth represents a critical phase in neurobehavioral maturation with rapid brain growth which corresponds to the last months of human fetal brain growth [26]. In our study, the development of the main parameters of physical maturation like eye opening, ear unfolding, incisor eruption, and hair appearance was not significantly different in all groups of animals. However, the development of sensorimotor reflexes important for the establishment of appropriate behavioral responses [68] was delayed in rats with prenatal hHCY. The day of appearance of negative geotaxis, righting reflex, cliff avoidance, and acoustic startle reflexes measured before P10 was slightly but significantly delayed in rats of the Hcy group. Reflexes which developed later and involved more complicated motor functions and different sensory systems were significantly delayed compared to the control group. Free-fall righting reflexes mediated by the visual, vestibular system, surface body senses, and proprioceptive senses appeared only at P19 (in control, at P12). The same delay was observed for cliff avoidance caused by visual stimuli and test olfactory discrimination, indicating variable development of different sensory systems. Similar observations were found in pups with gestational vitamin B deficiency where the implementation time of the negative geotaxis reflex was increased [8].

NaHS treatment not only improved the development of neurobehavioral reflexes in the Hcy group but even accelerated the appearance of the righting reflex and acoustic startle reflex in the control group which may be explained by the antioxidant properties of H_2_S and its contribution for healthy placental vasculature [53, 54]. Therefore, NaHS administration may accelerate the development of reflexes, as shown for the antioxidant agent Mexidol which administration during neonatal period facilitated learning processes of rats [69].

Exploratory behavior is typically assessed in an open field where the inner conflict of the animals to avoid potentially dangerous environments and eagerness to explore it determines their locomotion [70]. In our experimental approach, head rearing, number of crossed squares, rearings, and head dips in the open field test were significantly decreased in the Hcy group, indicating reduced exploratory behavior. Self-grooming behavior reflects the reaction of animals to a stressful environment [71]. Pups from the Hcy group showed an increase of grooming acts in the open field arena, which indicates higher stress susceptibility of animals. Most impressively, NaHS treatment restored all parameters recorded in the open field to the control level. Decreased exploratory behavior and high level of grooming in rats with prenatal hHCY observed in our experiments indicate on the depression and anxiety associated with hHCY conditions. These changes can be explained by decreased dopamine, serotonin, and norepinephrine levels and increased activity of monoamine oxidases in brain tissues [24, 72]. NaHS administration improved grooming and head dips in rats of the hHCY group, indicated its anxiolytic-like effect. Antidepressant and anxiolytic-like effects of H_2_S were previously shown in forced swimming and tail suspension tests of mice and rats—constituting behavioral models of depression and anxiety [73, 74]. In line with our results, H_2_S donor increased head dips and lowered the number of grooming of rats in the open field and elevated plus maze [75]. Possible mechanisms of H_2_S action include the inhibition of the corticotropin-releasing factor secreting from the hypothalamus under stress conditions [76, 77]. Recently, it was shown that H_2_S inhibits monoamine oxidase activity and restores concentrations of catecholamine and serotonin in the brain of rats with hHCY [24].

Hyperactivation of NMDA receptors with subsequent desensitization impacts on the impairments of brain maturation in prenatal hHCY [7, 8, 78, 79]. In addition, homocysteine increased activity of maxi Ca^2+^-activated K^+^ channels of rat pituitary tumor cells (GH3) and decreased growth hormone release necessary for growth and development [80]. H_2_S may prevent excitotoxicity associated with hyperactivation of NMDA receptors [39] as indicated by its inhibitory effects on GluN1/2B receptors, mainly expressed during the neonatal period preventing enhanced neuronal excitability typical for early hippocampal networks [81].

### 4.3. H_2_S Improves Motor Coordination and Muscle Strength of Rats with Prenatal hHCY

The paw grip endurance (PaGE) test demonstrated that at all tested ages (P4, P14, and P26), the time spent on the grid was lower in hHCY pups indicating diminished muscle strength. Moreover, the decreased latency to fall from the rotating cylinder and shorter rotarod distance indicated impaired fore and hind limb motor coordination and balance which may result from cerebellar dysfunction [82]. Also motor cortex, hippocampus, and basal ganglia play important roles in the performance of this task [83]. These brain areas accumulates homocysteine which induces oxidative stress with subsequent DNA damage and accelerated neuronal apoptosis in fetal brain [7, 84]. It was reported that hHCY conditions in *CBS*^+/−^ mice were detrimental to muscle force generation and responsible for muscle fatigability [85] via oxidative/endoplasmic reticulum (ER) stress [86]. Treatment of hHCY dams with H_2_S donor restored muscle strength, motor coordination, and balance of pups to control levels which may allude to the importance of endogenous production of H_2_S in rat skeletal muscle. Beneficial effects of H_2_S may be explained by the reduction of oxidative and ER stress responses in affected skeletal muscles [38, 86]. In addition, deleterious effects of homocysteine were shown at the level of the neuromuscular junction. Namely, it was shown recently that homocysteine depressed quantal content and largely increases the inhibitory effect of ROS on transmitter release, via NMDA receptors activation [87, 88]. Simultaneously, H_2_S increased quantal transmitter release in the mammalian neuromuscular junction [14]. Thus, a deficit of H_2_S production may be a plausible reason of muscle weakness observed in our study together with oxidative stress induced by hHCY.

## 5. Conclusions

We have shown that homocysteine-evoked oxidative stress during the prenatal period caused delayed brain maturation of the offspring and decreased H_2_S levels in brain tissues. Treatment of dams during pregnancy with H_2_S reversed the observed developmental impairments, restored muscle strength and coordination, and prevented oxidative stress of the brain tissue. Our data are supported by results obtained in models of acute hHCY in adult animals, where H_2_S obliterated homocysteine-induced endoplasmic reticulum stress as well as learning and memory deficits [22], ameliorated cognitive dysfunction, inhibited reactive aldehyde generation, and upregulated glutathione in the hippocampus [23]. Moreover, it was shown that endogenous H_2_S is required for healthy placental vasculature to support fetal development and that a decrease in CSE/H_2_S activity may contribute to the pathogenesis of preeclampsia [53]. Our findings suggest that H_2_S is effective in protection against developmental impairments in prenatal hHCY and has a promising potential role in facilitating a novel strategy to prevent homocysteine/oxidative stress-induced neurotoxicity.

## Figures and Tables

**Table 1:** The effects of NaHS treatment on the development of neurobehavioral reflexes of pups with prenatal hHCY.

Parameters	Control	Hcy	H_2_S	HcyH_2_S
Negative geotaxis (day of appearance)	6 (5–7) *n* = 55	6 (6–8)^∗^ *n* = 65	6 (6–6)^#^ *n* = 51	6 (5–7)^#^ *n* = 47
Head shake reflex (number of the head rotations per min at Р8)	7 (5–11) *n* = 53	2 (1–4)^∗^ *n* = 60	9 (4–14)^#^ *n* = 51	4 (2–6)^∗^^,#^ *n* = 47
Righting reflex (day of appearance)	6 (4–7) *n* = 55	6 (6–8)^∗^ *n* = 65	6 (3–6)^∗^^,#^ *n* = 51	4 (4–5)^∗^^,#^ *n* = 49
Righting reflex (time (s) at P6)	1 (1–2) *n* = 55	2 (2–4)^∗^ *n* = 65	1 (1–0.75)^#^ *n* = 51	1 (1–1.5)^#^ *n* = 49
Cliff avoidance test (day of appearance)	6 (5–7) *n* = 55	7 (6–8)^∗^ *n* = 60	6 (5–6)^#^ *n* = 51	4 (4–4)^∗^^,#^ *n* = 49
Acoustic startle reflex (day of appearance)	10 (8–10) *n* = 53	10 (9–11)^∗^ *n* = 55	8 (6–10)^∗^^,#^ *n* = 51	9 (8–12)^#^ *n* = 47
Cliff avoidance caused by visual stimulus (day of appearance)	14 (12–15) *n* = 53	16 (16–17)^∗^ *n* = 53	14 (13–16)^#^ *n* = 50	14 (14–15)^#^ *n* = 46
Free-fall righting (day of appearance)	12 (12–16) *n* = 53	19 (16–19)^∗^ *n* = 53	14 (13–14)^#^ *n* = 50	14 (13–14)^#^ *n* = 46
Test olfactory discrimination (day of appearance)	14 (12–15) *n* = 53	16 (14–19)^∗^ *n* = 53	14 (14–16)^#^ *n* = 50	14 (13–15)^#^ *n* = 46

Data are expressed as median (Q1–Q3). Statistical significance between medians was calculated using the nonparametric ANOVA Kruskal-Wallis test, Kolmogorov-Smirnov normality test, and Mann-Whitney. ^∗,#^*p* < 0.05. ∗ compared to the control group, # compared to the Hcy group. *n*: number of animals.

## Data Availability

The data used to support the findings of this study are available from the corresponding author upon request.
